# Facing life-threat during youth: a qualitative study on challenges, coping, and needs among adolescents and young adults with cancer

**DOI:** 10.1007/s00520-024-08370-0

**Published:** 2024-02-22

**Authors:** Carolin Wilharm, Anne Pralong, Mara Weiss, Michael Hallek, Raymond Voltz, Armin Tuchscherer, Steffen T. Simon

**Affiliations:** 1 Department of Palliative Medicine, Faculty of Medicine and University Hospital, University of Cologne, Kerpener Street 62, 50937 Cologne, Germany; 2 Department I of Internal Medicine, Faculty of Medicine and University Hospital, University of Cologne, Cologne, Germany; 3 Faculty of Medicine and University Hospital, Center for Integrated Oncology Cologne Aachen Bonn Cologne Duesseldorf (ABCD), University of Cologne, Kerpener Street 62, 50937 Cologne, Germany; 4 Faculty of Medicine and University Hospital, Center for Health Services Research (ZVFK), University of Cologne, Cologne, Germany

**Keywords:** AYA, Cancer, Life-threat, Challenges, Needs, Qualitative

## Abstract

**Purpose:**

While the unique situation of adolescents and young adults with cancer (AYAs) has become the focus of research and clinical practice, little is known about how they deal with the threat to life at a curative stage. The aim of this study was to obtain insight into the challenges, coping strategies, and needs of AYAs regarding the life-threatening nature of their diseases.

**Methods:**

Face-to-face in-depth interviews were conducted with patients who were 18–39 years old at diagnosis. The interviews took place 2–5 years after their diagnosis. Patients who were still undergoing treatment or who were suspected of recurrence were excluded. Interviews were transcribed verbatim and analyzed using qualitative content analysis.

**Results:**

Fifteen patients (mean age 27.33 years, nine females) were interviewed in a large comprehensive cancer center in Germany. Before diagnosis, AYAs had not faced their own mortality and had little experience with cancer. The sudden confrontation with a life-threatening disease and therapy, as well as experiencing the death of other AYAs, challenged them. Fear, particularly regarding recurrence and death, and the loss of trust in their own bodies were the major emotions that continued to limit them even after the end of treatment. For mothers, concern of leaving their young children alone was paramount. Coping strategies frequently mentioned were hope, avoidance, self-soothing, and valuing the experience as a chance. Health care professionals were expected to be reassuring, motivating, and open and to provide honest information based on individual and current needs.

**Conclusion:**

AYAs appear to cope with life-threats similarly to older patients but have additional unique challenges, including inexperience with life-threatening diseases and responsibility for young children. More research is needed in this area, although it is already evident that AYAs need honest and reassuring communication regarding the life-threat during any curable phases of their disease.

**Trial registration number** DRKS00030277; September 27, 2022 (German Clinical Trials Register).

## Introduction

In 2020, an estimated 4 million people in Europe were diagnosed with cancer in 2020 [1]—a disease that is universally associated with the thought of death [2]. What this means to patients has been well studied, especially in psycho-oncology and palliative care [3–6], and interventions have been conceived to support them [3].

This research refers mainly to elderly people. However, in 2020, approximately 112,000 15–39-year-olds in Europe were diagnosed with cancer, and incidence rates in this age group are increasing [7].

Adolescents and young adults with cancer (AYAs) differ from older patients [8, 9]. They face a unique life situation with various transitions and uncertainties like completing education or becoming a parent [10], which may affect their challenges, coping strategies, and needs when diagnosed with cancer. Studies have shown that cancer diagnoses significantly impact on AYAs’ quality of life and can lead to the development of psychosocial distress and mental disorders [11, 12].

Their specific challenges and needs caused by cancer have long been neglected, but interest in this topic has grown recently, and tailored supportive programs already exist in various countries [9, 13].

Challenges and needs caused by the life-threat have been examined in-depth predominantly in AYAs with incurable diseases yet [14–16]. However, the threat to life can be challenging for AYAs at all stages of cancer [17, 18]. A mixed-methods study by Hølge-Hazelton showed that over 80% of AYAs surveyed were concerned with fear of death, and many were affected by it in their daily lives, often in far-reaching ways [17]. Discussions about this rarely take place or only occur at the end of life [14, 17]. There is a lack of research on this issue for AYAs in a curative stage of their disease [14].

To fill this research gap, we aimed to interview AYAs between 18 and 39 years of age at diagnosis of cancer 2–5 years after diagnosis, when they had completed their therapy, to gain in-depth insights into their challenges, coping strategies, and needs regarding the life-threatening nature of their disease.

## Methods

### Study design

A narrative interview study [19] was conducted with AYAs 2–5 years after their diagnosis. Participants had to have completed their therapy. This allowed us to gain insight into the overall duration of therapy and vulnerable time points during it. Results not related to life-threat will be published separately.

The study was approved by the Ethical Review Committee of the Medical Faculty of the University of Cologne (Ref.Nr. 22–1105) and is registered in the German Clinical Trials Register (DRKS00030277). The reporting of this study has been conducted according to the “Consolidated Criteria for Reporting Qualitative Research “ (COREQ) [20].

### Participants

We included patients aged 18–39 years at initial diagnosis. The deviation from the predominant age definition of 15–39 years [13, 21] was chosen, in concordance with other German AYA studies, because minors in Germany are treated mainly by pediatricians and thus differ greatly from older AYAs [22].

In addition to the heterogeneity of participant characteristics, we aimed to recruit patients with the types of cancer most prevalent in young adulthood.

Additional inclusion criteria were: first cancer diagnosis ≥ 2 years and ≤ 5 years before study participation, completion of oncologic treatment (except maintenance therapy), underlying oncologic disease in remission/cured at the time of study participation, and written informed consent.

Patients who could not be interviewed due to cognitive impairment or lack of German language skills and those with (suspected) recurrence were excluded.

### Recruitment

Participants were recruited from June 2022 to April 2023 via the Center for Integrated Oncology (CIO), one of the largest cancer centers in Germany, and the University Hospital of Cologne.

After obtaining consent from the treating physician, the interviewer (CW), a female research associate and physician of similar age as the participants, informed the patients about the study via e-mail, telephone, or in person at a follow-up appointment. If they were interested, they were given an information letter, and an appointment was made. A written informed consent was signed by all participants prior to interviews. None of the participants were known to the interviewer prior to the study.

Recruitment was terminated when theoretical saturation was achieved, that is, when no new information were gained in further interviews [23].

### Interviews

The interviews were conducted in consulting rooms of the CIO; a duration of 60–90 min per interview was scheduled.

The participants were allowed to bring a confidant with them, to stop the interview at any time, or/and to seek psychological help. The interviewer was supervised regularly.

Before beginning, the interviewer (CW) again introduced herself and the study and answered any open questions.

The interviews were semi-structured (see Table 1) to obtain uninfluenced information to the greatest degree possible and to discuss topics relevant to the participant adequately. Repeat interviews were not conducted.

**Table 1:** Interview guide (part: life-threat)

Major theme	Sample questions
Introduction	Name, profession, functions of the interviewer Interview procedure and aims “You can interrupt or cancel the interview at any time and request psychological support.” “Do you have any further questions before we start?”
Experiencing the life-threat	“When did you become aware of the threat to your life?” “What triggered this in you?”
Dealing with the life-threat	“How did you deal with the threat to your life?”
Needs regarding to the life- threat	“What support did you receive to help you cope with the threat to your life?” “What helped you?” “What would you have wished to have?”
Open-ended closing question	“Are there any other topics/aspects/etc. that have not been mentioned so far?”

Key patient characteristics, taken from the patient files or requested from the patient before the interview, were documented.

### Analysis

The interviews were audio-recorded, anonymized, and then transcribed verbatim using the MAXQDA software (2022, VERBI GmbH, Berlin, Germany).

Subjective, especially non-verbal, impressions of the interviewer were included in the analysis. Transcripts were not returned to the patients.

The transcripts were analyzed using content analysis according to Kuckartz using MAXQDA [24]. All interviews were initially analyzed by CW, creating a coding framework. The main categories (challenges, coping, needs) were formed deductively based on the research question. All other categories were derived from the data [24].

To increase the objectivity of the coding process, all interviews were additionally coded by a medical student [24, 25]. Conflicting results were discussed with a senior consultant (StS and AT). Finally, the categories were analyzed, especially regarding similarities and differences. Results were critically discussed, until consensus was reached.

## Results

### Characteristics of the study population

A total of 28 patients were invited to participate; seven refused, and six did not report back. Reasons for refusal were lack of time (four patients) and personal reasons (two patients); one patient gave no reason. Fifteen patients consented and were included in the study.

Saturation was already evident after 13 interviews; therefore, recruitment was terminated after 15 interviews. Interviews lasted on average ca. 50 min (23–66 min). All patients were interviewed alone, except for one who brought his 10-month-old child.

For more detailed information about patient characteristics, see Table 2.

**Table 2:** Patient characteristics

CoVariate	Number
Participants	15
Gender (f/m/d)	
f	9
m	6
Age at initial diagnosis (mean 27.33 years + / − 6.07 years)	
18–28 years	7
29–39 years	8
Age at interview (mean 30.07 years + / − 5.92 years)	
18–28 years	7
29–39 years	8
Months since initial cancer diagnosis (mean 42 months + / − 10 months)	
24–35 months	4
36–47 months	4
48–59 months	7
Cancer diagnosis	
Hodgkin lymphoma	4
Acute lymphocytic leukemia/acute myeloid leukemia	3
B-cell-lymphoma	2
Germ cell tumor	2
Thyroid carcinoma	1
Colon carcinoma	1
Breast cancer	1
Ewing’s sarcoma	1
Cancer-related therapy	
Chemotherapy	4
Chemotherapy + radiotherapy	5
Chemotherapy + allogeneic stem cell therapy	2
Surgery + chemotherapy + radiotherapy	1
Surgery + chemotherapy + radiotherapy + antihormonal therapy	1
Surgery	2

### Challenges

#### Sudden confrontation with their own mortality

Existential concerns were not prevalent for the AYAs before diagnosis. They had not yet come to terms with their own mortality. For the most of them, they had never known anyone, especially someone of their own age, who had been diagnosed with cancer. Accordingly, the diagnosis of a life-threatening disease was a shock.

“Before the diagnosis, honestly, I didn’t think about dying. So, you’re young, what’s going to happen.” (P6, m, 29 y, seminoma)

“Suddenly somehow to have such a diagnosis: Yeah, maybe your life will end sooner than you think, that was kind of hard [...] that I thought: oh shit, not that it’s over now before it even really starts.” (P15, f, 33 y, breast cancer)

#### Additional life-threat via therapy

Before diagnosis, AYAs knew little about cancer treatment. The fact that not only the disease but also its therapy is life-threatening presented a further challenge after diagnosis. Side effects and their potential extent had a strong impact on AYAs.

“I was just new to the topic cancer [...] I had to read through all that first and then in the course of that I first got to know a little bit the, yeah, the extent of the disease.” (P11, m, 24 y, Hodgkin’s lymphoma)

“[The doctor] told me directly during the first consultation that it is a life-threatening therapy. And I always thought that the diagnosis was life-threatening, and she said that the therapy was also life-threatening. And that totally dragged me and my boyfriend down at that moment […]” (P3, f, 30 y, NHL)

#### Experiencing the deaths of other AYAs

In general, the AYAs regarded exchanges with each other as helpful. However, it affected them badly when they had to witness others’ deaths. On the one hand, they grieved for the person; on the other, they became immediately aware that they themselves could die from the disease.

Such an experience or the fear of it sometimes led AYAs to retreat from others.

“And there was the girl I met here […] who said: Yes, she has now no further treatment options, nothing more can be done, she won’t make it. And that was the moment when I said [...] that’s too close to me. Then to hear about others who are my age [...] Yes, I think that was one of the most difficult things, these fates of so many.” (P5, f, 23 y, sarcoma)

#### Fears

*Fear of death* in various forms and manifestations was an important issue in the interviews. While some AYAs considered death very improbable, others were firmly convinced that they would die, especially in the early stages. The extent of fear fluctuated during the therapy process and could become stronger as a result of the challenges described above.

“And life-threat somehow always floats along still a little.” (P9, f, 36 y, colon carcinoma)

“Because I kind of thought I was going to die in surgery. I don’t know why I thought that, so somehow it was clear to me: I’m not going to wake up.” (P9, f, 36 y, colon carcinoma)

*Fear for their own children*. Both mothers interviewed at the time of diagnosis were very concerned about the possibility of dying. The impact this would have on their young children and the thought that their time with them might be very limited caused them great distress.

“[…] all of a sudden it was somehow in the room that I might not get to see my children starting school [...] [starts crying harder, continues speaking haltingly]. So, that was just, yeah, very scary, that knocked me out a lot.” (P8, f, 36 y, Hodgkin’s lymphoma)

*Fear of recurrence*, which persisted years after the end of treatment, was mentioned frequently. It was particularly present before follow-up appointments and lead to uncertainty and sometimes sleep disturbances.

Patients were aware that the survival probability in case of a recurrence would be significantly lower. Added to this was the knowledge about the treatment effects.

“[…] you always have a certain residual fear inside you that what you once had will come back at some point and then the chances are just worse. [...] this permanent fear that something could happen again at some point and that you then have to go through exactly what you’ve already gone through.” (P13, m, 23 y, AML)

#### Loss of trust in their own bodies

Many participants reported a feeling of vulnerability and that they had lost trust in their bodies, which would take time to rebuild. This related not only to cancer but also to other diseases and general situations and therefore had a far-reaching impact on their entire lives. For example, one patient reported that she had lost the confidence to make longer journeys.

“It’s such a conflict that I just think, ‘Okay, I’m fine,’ but on the other hand, I’ve also completely lost this physical relationship, this health relationship to my body, and I just no longer have this trust in what I used to have, or where one lived so lighthearted.” (P2, f, 19 y, NHL)

#### Death becoming more real afterwards

Compared to before the illness, most participants felt that death was more real afterwards. By this, they meant not only their own death but also that of their relatives.

“I’m still afraid of dying in general, and it’s just become a little more present that it’s just part of life at some point.” (P8, f, 36 y, Hodgkin’s lymphoma)

“So, it’s more like actually maybe I worry more about other people dying; my parents are getting older and that’s something I’m afraid of.” (P4, m, 29 y, AML)

### Coping strategies

#### Hope and optimism

Hope for healing and an optimistic attitude were mentioned very frequently. These issues related closely to avoidance, as patients tried to put thoughts of healing above those of dying. Hope persisted continuously, even simultaneously with thoughts of death. In some cases, the patients themselves were surprised at how little they doubted a positive outcome.

“So, I never didn’t have courage, it wasn’t now that I ever kind of doubted there that I was going to make it. It was kind of an amazing optimism.” (P4, m, 29 y, AML)

“[…] on one side the consciousness came through or the knowledge came through: Yes, it can lead to death. While from the other side, somehow at the same time, there was simply this hope and also the thought: Well, that won’t happen to you. So, exactly. That, yes, that kind of clashed a little bit in me.” (P15, f, 33 y, breast cancer)

#### Avoidance

In addition to concentrate on healing, participants also tried to repress thoughts about death as much as possible, in some cases by actively fighting them. This behavior continued beyond the therapy and was predominantly evaluated as a good protective mechanism.

“I kind of pushed it away a little bit because I think [...] if I had been too concerned with the fact that I could have died, then I wouldn’t have, I wouldn’t have gotten through the whole thing the way I did.” (P9, f, 36 y, colon carcinoma)

#### Self-soothing

In contrast to avoidance, some patients found a method of self-soothing in actively dealing with possible death. In moments when life-threat became present and frightened them, they repeatedly told themselves that they could accept dying. They listed arguments why death would be acceptable despite their young age. At the same time, they were aware that this option, should it become real, would be very challenging for them.

“If I tell myself, it’s okay now, even if I were to die … that actually calms me down sometimes.” (P2, f, 19 y, NHL)

#### Life-threat as a chance for a future life

Several reported that confronting their own mortality had led to rethinking behaviors and views. They described that they would appreciate life more now.

“That’s such a lesson that—kinda this being confronted with my own mortality—also just kinda guides my actions a little bit now.” (P15, f, 33 y, breast carcinoma)

“[…] I am now just more aware that I am mortal. Which I think is a good thing [...] because that has also led to me, for example, becoming braver somehow or thinking more like, I’m doing things now, I’m not putting it off until later.” (P10, f, 33 y, thyroid carcinoma)

### Needs

#### Need to focus on hope

The AYAs wished for conversations with health care professionals (HCPs) to focus on their hope for a cure for the cancer. They found the emphasis on good chances for being cured, if they existed, helpful. They also appreciated doctors who provided reassurance and a relief of fears. This helped them to feel safe.

“[Name of the doctor] was also extremely helpful in this, [...] who then somehow radiates such extreme calm and then also doesn’t let you panic.” (P13, m, 23 y, AML)

“Because the doctor said I’m not going to die, so I’m not.” (P7, f, 18 y, Hodgkin’s lymphoma).

#### Need (not) to discuss life-threat

To keep their positive attitude and be able to suppress the issue, the participants wished that the life-threat was not mentioned too often, particularly not in every conversation.

“I thought it was good that it wasn’t addressed because that’s what kept me in that positive mindset.” (P1, f, 18 y, ALL)

One instance when the life-threat should be addressed was stated as after the diagnosis and when there was bad news about prognosis. Because despite the focus on healing, open and honest information handling was considered important.

“And at the same time [...] a never dishonest, but still always kind of motivating, posi … slightly positive vibe that was given to you.” (P13, m, 23 y, AML)

#### Need to be listened

In certain situations, such as a change in therapy, some AYAs had the need to talk but did not always express this clearly. Therefore, they found it important that the HCPs signaled openness for conversations and questions. Taking time was seen as crucial. Empathy was stated as an important competence and attitude for physicians.

“It’s always good to talk and to always have the opportunity to [...] ask someone who is familiar with the therapy [...] about everything. That was always important to me personally, to be able to ask really stupid questions.” (P4, m, 29 y, AML)

#### Need for information

Wide variation existed regarding how much information patients wanted to receive. While some did not want to hear “horror scenarios” (P5, f, 23 y, sarcoma), others wanted to know “what is the worst thing” (P7, f, 18 y, Hodgkin lymphoma).

Participants needed explanations in a comprehensible form. If they understood what was happening to them, fear could be lessened.

“I think [...] giving a sense of ‘We know what to do, we’ll guide you through it, and if you want, we’ll also explain everything to you in as much detail as you need’ is quite important.” (P15, f, 33 y, breast cancer)

#### Need for reassurance through aftercare

When AYAs were told at follow-up appointments that no evidence of recurrence was present, the fears described above decreased immediately and recurred only slowly. Participants wished for timely appointments when they suspected that the cancer might have returned.

“Because I also have more with each examination, where you get a result, [...] that everything continues to be good […], that already increases the confidence again. And also somehow gives you peace of mind for the next few months, until the next examination.” (P14, m, 26 y, seminoma). The results are summarized in (Table 3).

**Table 3:** Summary of categories

Before diagnosis	After diagnosis		
	Challenges	Coping	Needs
- death not in mind - no experiences with life-threatening diseases among friends/family - little knowledge about cancer (therapy) - caring for young children	- sudden confrontation with their own mortality - additional life-threat via therapy - experiencing the deaths of other AYAs ►fears ►loss of trust in their own bodies ►death more real afterwards	- hope and optimism - avoidance - self-soothing - life-threat as a chance for a future life	- to focus on hope - (not) to discuss life-threat - to be listened - for information - for reassurance through aftercare

## Discussion

To the best of our knowledge, this is the first interview study about life-threats’ impact on AYAs with a curative disease. The interviews revealed that the sudden confrontation with their own mortality could affect AYAs both positively and negatively, in terms not only their disease but their entire lives.

The interviewed AYAs developed various, often simultaneously existing coping strategies, such as hope, avoidance, and self-soothing. They wished for their coping to be supported by HCPs without concealing relevant information, such as prognoses.

The majority expressed a fear of dying, yet many did not need to talk about it. The time following the diagnosis and a loss of trust in their own bodies were described as particularly stressful. Hølge-Hazelton et al. described similar results in their survey of 822 AYAs [17].

In contrast to other AYA studies, maladaptive coping strategies, such as substance abuse [26], were not reported by our participants. This may be due to the open question, which allowed participants to avoid this topic.

Comparing our results and those of other AYA studies [27, 28] with studies of older cancer patients, the coping strategies of AYAs appear to differ little from those of older patients. Hope and optimism, for example, are described as helpful in many studies with cancer patients of all ages[29–31]. They have a positive relationship with quality of life [31] and can reduce distress [29, 31].

Concurrently, our participants preferred honest information about prognosis. Studies with older patients [32, 33], as well as AYAs with advanced cancer [15], confirm this.

The fear of recurrence is also commonly reported in the literature, occurs independent of age, and is perceived as very distressing [34–36]. The time just before a medical appointment is described as a trigger [35, 36]; therefore, the reassurance provided by this is perceived as helpful [35].

Nevertheless, additional challenges arise from the unique life situation of AYAs. Especially among young AYAs, the unexpectedness of the diagnosis and their lack of knowledge about the disease are prevalent [37], as was also shown in our interviews. They often do not know anyone who had had cancer before and know little about the therapy. They have usually not yet dealt with their own mortality. Studies have shown that a peers’ death is exceptional imprinting for young adults [38–40] and underline the deep impact of dying and death at that age.

Although older patients are also concerned about their relatives [41], this is a different dimension for AYAs when they have the responsibility for young children. AYAs are aware of their death’s great impact on their families’ futures.

Some participants drew benefits for their future from the life-threat by rethinking their previous lives. This common behavior can increase quality of life [42, 43]. However, it has a stronger impact on AYAs who still have most of their lives ahead of them and for whom this can influence crucial decisions.

### Strengths and limitations

This study has several limitations. It is not representative because the number of participants is small, as is usual in qualitative research, and was recruited in only one center. It is possible that COVID-19 measures impacted the treatment and interviewing of the participants.

However, it provides in-depth insights into the participants’ challenges, coping strategies, and needs for support regarding life-threat in a curative state of their disease. Despite the small number of participants most of the frequent diagnoses could be represented in a heterogeneous study group.

## Conclusion

Participants faced fears stemming from sudden existential concerns while having little knowledge about cancer and its therapy. Although our results indicate that many similarities exist between AYAs and older patients in dealing with the life-threat, differences must be considered and further researched. AYAs should receive the best possible support for their coping strategies from HCPs. An optimistic attitude should be fostered and fearful patients should be reassured without withholding desired information. It is particularly important to listen to them, explain, and inquire whether there is a need for support. Further research is needed to improve the understanding of AYAs’ challenges and dealing with the life-threat when their cancer is curable.

## Data Availability

The datasets generated during and/or analyzed during the current study are not available for data protection reasons.
